# Draft genome sequence of a *spa* non-typeable *Staphylococcus aureus* USA300 isolate causing complicated bacteremia

**DOI:** 10.1128/mra.00701-24

**Published:** 2024-09-09

**Authors:** Emily A. Felton, Jesus Diaz Vera, Ju Hee Katzman, Sara Daniels, Amorce Lima, Deanna Becker, Suzane Silbert, Kami Kim, Lindsey N. Shaw

**Affiliations:** 1 Department of Molecular Biosciences, University of South Florida, Tampa, Florida, USA; 2 Center for Antimicrobial Resistance, University of South Florida, Tampa, Florida, USA; 3 Division of Infectious Disease and Internal Medicine, University of South Florida, Tampa, Florida, USA; 4 College of Public Health, University of South Florida, Tampa, Florida, USA; 5 Tampa General Hospital, Tampa, Florida, USA; 6 Center for Global Health Infectious Diseases Research, University of South Florida, Tampa, Florida, USA; 7 Global Emerging Diseases Institute, Tampa General Hospital, Tampa, Florida, USA; Rochester Institute of Technology, Rochester, New York, USA

**Keywords:** *Staphylococcus aureus*, bacteremia, USA300, clinical isolate

## Abstract

Spa-typing is a major genetic tool used to distinguish between *Staphylococcus aureus* strains. Interestingly, although rare, ~1%–2 of isolates are considered Spa-non-typeable . Herein, we present the draft genome sequence of just such a strain, *S. aureus* TGH1097, a USA300 isolate from a complex bacteremia infection.

## ANNOUNCEMENT

A patient presented at Tampa General Hospital with gradually worsening 3-week duration subacute productive cough, fatigue, shortness of breath, and mid-thoracic back pain. The patient was septic and complicated by mitral valve infective endocarditis, multifocal cavitary septic pulmonary emboli, large prevertebral empyema with adjacent osteomyelitis and discitis, and combined dorsal/ventral spinal epidural abscess. Blood was collected from the patient via a right antecubital draw and plated on both blood agar and MacConkey agar. These grew a methicillin-resistant *S. aureus* isolate (TGH1097), which was taken from the blood agar plate and subcultured on blood agar to ensure purity. For bacterial DNA extraction and whole genome sequencing, TGH1097 was grown overnight (37°C, 250 rpm) in tryptic soy broth (BD, Heidelberg, Germany). Genomic DNA was extracted using the Qiagen DNeasy Blood and Tissue kit with 20 mg/mL lysostaphin added to the enzymatic lysis buffer. DNA quality and quantity were assessed using a Quant-it fluorescent assay kit (Invitrogen) with a Biotek Citation5 microplate reader. An Illumina Nextseq2000 instrument with a Hackflex (1) adapted version of Illumina DNA-seq library preparation kit and P1 flow cell chemistry produced 178.9 Mbp (1,184,965) of paired-end 2 × 150-bp reads (>63× idealized coverage). The raw reads were trimmed via Trimmomatic v0.39 (2) with SLIDINGWINDOW:10:20 MINLEN:31 TRAILING:2 settings (3). Trimming quality checks were performed with FastQC v0.12.0. The reads were then *de novo* assembled with SPAdes v3.15.5 default settings (4), and the assembly was checked for quality via Quast v5.2.0 (5). The TGH1097 genome assembly was generated from 1,176,774 reads to yield 122 contigs with a total length of 2,931,580 bp and mean coverage of 62× depth (*N_50_* 283,796 bp; largest contig, 600Kbp). Within our contigs, fragments of the multidrug-resistant plasmid pUSA04 were detected from reads with high coverage bias. The overall G + C content of the genome was 32.63%. The Staphtyper workflow that is part of the Bactopia v3.0.1 characterized TGH1097 as Agr group 1 (6), SCCmec type IVa (7), but was unable to assign a known Spa-type (8) to the identified repeats (04–21-17-25 04–21-17-25). Review of the *spa* ORF revealed 50 amino acids in frame deletion in the Xr region, which consists of a variable number of octapeptide repeats used for *spa* typing (9–14). This thus explains the *spa* non-typeable nature of TGH1097.

## Data Availability

The full sequencing project’s raw reads are available in SRA study SRP514572; the TGH1097 BioProject accession is PRJNA1125331, with BioSample SAMN41893242 and SRA accession number SRS21665968. This Whole Genome Shotgun project has been deposited at DDBJ/ENA/GenBank under the accession JBETKU000000000. The version described in this paper is version JBETKU010000000.
